# Editorial: Exploring the interaction between health-promoting and health risk behaviors in health, volume III

**DOI:** 10.3389/fpubh.2026.1970474

**Published:** 2026-09-02

**Authors:** Feng Jiang, Huixuan Zhou, Yi-lang Tang

**Affiliations:** 1 School of International and Public Affairs, Shanghai Jiao Tong University, Shanghai, China; 2 Institute of Healthy Yangtze River Delta, Shanghai Jiao Tong University, Shanghai, China; 3 Department of Physical Fitness and Health, School of Sport Science, Beijing Sport University, Beijing, China; 4 Key Laboratory of Exercise and Physical Fitness, Ministry of Education, Beijing Sport University, Beijing, China; 5 Department of Psychiatry and Behavioral Sciences, Emory University School of Medicine, Atlanta, GA, United States; 6 Mental Health Service Line, Atlanta VA Medical Center, Decatur, GA, United States

**Keywords:** health equity, health governance, health literacy, health-promoting behavior, health-risk behavior, proactive health

Health emerges from the dynamic interplay between health-promoting and health-risk behaviors in everyday life (1). The 47 contributions to this Research Topic emphasized the complexity of this process, showing that health-promoting and health-risk behaviors often coexist in the same individuals, sometimes clustering together and sometimes unfolding along partly independent pathways. Taken together, these studies addressed three interconnected questions: how do health-promoting and health-risk behaviors interact? How do individuals develop the capacity to navigate and manage these behaviors? How do social and institutional contexts support the maintenance of healthier actions over time?

Several contributions examined behavioral patterns and trajectories. Sun et al. identified risk behavior clusters among older adults, while Wang Q. et al. examined multiple movement, sleep, screen-related, and other behaviors simultaneously in relation to college students' mental health. Kang et al. reviewed the related yet partly independent roles of physical activity and sedentary or screen-based behavior in adolescent health. Using a 24-h guideline framework, Wang et al. examined the relationship between moderate-to-vigorous physical activity, screen time, and sleep in relation to non-suicidal self-injury. Wu and Wan found that positive and negative communication legacies followed asymmetric pathways, with the positive pathway being stronger in recurring epidemics than in emerging epidemics. Together, these studies argue for attention to the combinations individuals actually experience.

Digital behavior provides a particularly revealing example of how risks and protective practices become intertwined. Huang et al. found that higher autonomous participation in physical activity was associated with weaker links between internet addiction, upward social comparison, and appearance anxiety. Xu and Li reported longitudinal interrelations among mobile phone addiction, bedtime procrastination, and physical activity. Together, these findings place problematic digital use within a broader behavioral pattern involving social comparison, bedtime procrastination, and lower physical activity. Effective responses need to address the motives and routines that connect these behaviors. Opportunities for autonomous physical activity and practical support for self-regulation may help individuals establish healthier routines.

This Research Topic also asked what enables individuals to act. Volume II proposed a proactive health model (2), while Volume III clarified what that model asks of health governance. Proactive health is the continuing ability to anticipate health needs, initiate action, monitor changes, seek help, and adjust behavior in response to credible feedback. This view is consistent with the contemporary definition of health promotion as a process that enables greater control over health (3). Wang, Pan et al. associated proactive behavior among subjects at high risk of stroke with health literacy, self-efficacy, risk perception, and chronic illness resources. Jung et al. linked health literacy with health-promoting behavior among college students with different levels of sports participation. Yang H. et al. reported associations involving social support and perceived control among surgical patients with lung cancer, and Yu et al. identified indirect associations through subjective wellbeing and self-efficacy among adults with chronic diseases. Across these populations, the studies consistently associated health-related action with knowledge, confidence, resources, and relationships.

This evidence changed how responsibility should be assigned. Health literacy helps individuals recognize risk, but it has limited practical value when services are difficult to navigate. Self-monitoring may detect deterioration, but a care pathway must be available when professional assistance is needed. Public support, in turn, depends on personal participation. Proactive health is best treated as supported agency, wherein individuals initiate and sustain action, while institutions make healthy action intelligible and durable.

Individual capacity, however, is exercised within broader social and institutional contexts. The evidence in this Research Topic spans households and neighborhoods, schools and peer networks, and the design and implementation of health programs. Li and Zheng found a rural-origin disadvantage in life satisfaction among graduate students at an elite university; measured health behaviors accounted for approximately half of the gap, while the remaining difference pointed to potential structural disadvantage. In interviews with urban Indian young adults, Rathi et al. identified family influence, time constraints, food environments, peer pressure, and the features of the built environment as practical determinants of healthy living. In school settings, Li L. et al. found that a supportive sports climate was associated with greater exercise self-efficacy and lower social anxiety. Long et al. used network modeling to show how centrality-based peer targeting could accelerate the modeled diffusion of physical activity, while leaving socially peripheral adolescents harder to reach. At the implementation level, Alsaqqa and Alwawi reviewed contextual and implementation factors relevant to the scale-up and sustainability of physical-activity interventions.

These findings align with research on the commercial determinants of health (4) and the WHO Commission on Social Determinants of Health report (5), both of which emphasize that opportunities for health are shaped by broader distributions of resources, power, and access. National and local authorities influence these opportunities through the regulation of harmful products and the design of transport, recreation, food, and digital environments. Schools, workplaces, primary care services, and community organizations mediate how these arrangements are encountered in everyday life. Ultimately, accountability can be assessed through a simple question: once a person recognizes a health risk, can they readily access and complete the next appropriate action?

This perspective favors interventions that address combinations and trajectories of behavior rather than isolated risk factors. Support is especially important during key life transitions, including adolescence and the transition to university, employment, or independent living. Health services and digital systems should connect self-monitoring with timely professional advice and accessible care pathways. Digital tools also need safeguards for privacy, transparency, accessibility, and human oversight, together with a clear route for escalating alerts to actual services. Support should be proportionate to barriers and risk because identical messages do not create an equal capacity to act.

Much of the evidence in this Research Topic remains observational, making longitudinal studies, causal designs, and multilevel intervention research priorities for the next stage. Evaluation should extend beyond behavioral and clinical outcomes to include reach, burden, trust, sustainability, and distributional equity. Collectively, the contributions in Volume III advance the idea of proactive health, shifting it from a personal aspiration to a principle of health governance. Humans are active producers of health, but they do not act alone. The central message emerging from this Research Topic is that healthier lives are sustained when personal initiative is supported by environments, public institutions, and policies that make healthy choices feasible, accessible, and enduring. Proactive health governance can succeed only when individual initiative is translated into durable action, and public institutions are held accountable for creating the conditions that make such action possible.
